# Regulatory roles of eugenol in paraquat-altered SNCA/LZTS3/MAPT in the cerebellum of Wistar rats

**DOI:** 10.1186/s42826-025-00236-8

**Published:** 2025-01-15

**Authors:** Obinna Onwe Uchewa, Augustine Oseloka Ibegbu, Samuel Okoronkwo Okafor, Joseph Alo Nwafor, Ogugua Augustine Egwu

**Affiliations:** 1 Anatomy Department, Faculty of Basic Medical Sciences, Alex Ekwueme, Federal University, Ndufu-Alike, Ebonyi State Nigeria; 2 Anatomy Department, Faculty of Basic Medical Sciences, David Umahi Federal University of Health Sciences, Uburu, Ebonyi State Nigeria

**Keywords:** Cerebellum, Eugenol, Limb functionalities, LZTS3, MAPT, Paraquat, SNCA

## Abstract

**Background:**

The Microtubules-associated protein tau (MAPT), alpha-synuclein (SNCA), and leucine zipper tumor suppressor 3 (LZTS3) genes are implicated in neurodegeneration and tumor suppression, respectively. This study investigated the regulatory roles of eugenol on paraquat-altered genes.

**Results:**

Forty male Wistar rats divided into five groups of eight rats were used. The control group received normal saline; the Paraquat (PQ)-untreated group received only Paraquat. The low dose of eugenol was 200 mg/kg, the medium dose of eugenol was 400 mg/kg, and the high dose of eugenol was 600 mg/kg. All groups except the control group received 10 mg/kg of PQ orally for 14 days at one-day intervals, allowing PQ in the rats for 28 days. Eugenol treatment started on the 29th and lasted 14 days. Motor impairments were determined using wire string and beam-walk; biomarkers were estimated using cerebellar homogenates, while frozen cerebellum was used to study LZTS3, MAPT, and SNCA gene expression. LZTS3 was significantly suppressed in the PQ-untreated group and highly expressed in the eugenol-treated group. The MAPT and SNCA genes were overexpressed in the PQ-untreated group compared to the control group. Eugenol significantly decreased the expression of these genes compared to that in the PQ-untreated group. Antioxidants were reduced considerably, and oxidative stress markers were increased significantly, which could have caused increased protein fibrillation and reduced limb functionality. Histology revealed that eugenol mitigated the alterations caused by Paraquat.

**Conclusions:**

PQ can enhance tumor expression in addition to causing neurotoxicity, which decreases limb functionality, while eugenol, an antioxidant, can mitigate the effects of PQ.

## Background

Environmental factors have been proven to cause genetic variation, directly increasing neurodegeneration susceptibility [1]. Parquat, a neurotoxicant, has been implicated in neurodegenerative diseases like Parkinson’s disease (PD) and Alzheimer’s disease (AD). The exact mechanisms by which Paraquat causes neurodegeneration are still being investigated, but relevant changes brought about by its exposure include elevated oxidative stress, dysfunctional mitochondria, protein accumulation, excitotoxicity, and autophagy [2, 3]. These modifications also change the miRNA expression profile, which in turn causes neuronal [2, 4]. According to a new study published in The Lancet Neurology, 2024, over three billion individuals were living with neurological disorders in the world in 2021, making it the leading cause of diseases and impairments globally [5, 6]. Since 1990, the total number of disabilities, ailments, and early death caused by neurological conditions has risen by 18%, as reported by the World Health Organisation [6]. More than 80 per cent of neurological deaths and health impairments are recorded in low- to middle-income nations, and there are significant differences in the availability of care: high-income nations have as many as 70 times more neurologist specialists per individual than do low- and middle-income nations [6].

The emergence of novel genomic technologies facilitates the identification of specific genes responsible for numerous neurologic disorders with a radically fresh perspective on their aetiology [7]. These developments and recent advances in gene therapy are starting to translate a person’s genetic makeup into personalized treatment plans, revolutionizing the knowledge of the mechanisms behind brain illnesses [8]. Novel techniques and analytical frameworks, such as genome array research and next-generation sequencing technologies, provide a greater understanding of the minute details of the genetic architecture that controls susceptibility to specific ailments [9]. The extent to which gene studies have revealed certain disorders and fundamental neurobiology is astonishing [10]. According to Koenig et al. [11], who pioneered the discovery of the Duchenne muscular dystrophy (DMD) gene by Hoffman, [12], Gu et al. [13] and Haddadi et al. [7], this gene plays a vital role in clinical genetics, cytogenetics, and linkage in determining gene locations. Following the discovery of DMD as the first gene for an inherited disorder, it has been used as a model disorder for developing new diagnostics and therapeutics for a genetic disorder [14].

However, the discovery of dinucleotide polymorphic repeats and the simplicity of genotyping with polymerase chain reaction (PCR)-aided genetic mapping have revolved around studying genetic diseases [15, 16]. The tau protein is an abundant protein in the brain whose autosomal dominant mutation, the MAPT gene, was discovered in 1998 as the cause of certain diseases [17, 18]. The malfunction of tau is sufficient for widespread dementia [19, 20] and cognitive, behavioural, and motor impairments [21]. As of 2018, approximately 50 distinct pathogenic missense, silent, and intronic MAPT mutations have been identified [18, 22]. The leucine zipper tumour suppressor 3 (LZTS3) gene is found in proteins that regulate gene expression during cell differentiation and tumour growth [23, 24]. The mechanism underlying LZTS3 gene-mediated tumour development inhibition is still unclear [25, 26]. Most studies on LZTS3/ProSapip1 have focused on the nervous system [27, 28]. The LZTS3 gene may be regulated by the upstream gene mTORC1, resulting in altered actin expression [29–31]. The SNCA gene encodes alpha-synuclein [32], a neuronal protein that modulates synaptic vesicle transportation and the release of neurotransmitters [33]. The protein is prevalent in the brain, with lower levels found in the heart, muscles, and other organs, and predominant in presynaptic neuron axon terminals [34]. The SNCA interacts with proteins and phospholipids [35] at these junctions [36]. Presynaptic terminals release chemical messengers and neurotransmitters from synaptic vesicles, essential for regular brain functioning [32]. In Parkinson’s disease and other synucleinopathies, insoluble forms of alpha-synuclein aggregate as inclusions in Lewy bodies [37]. Eugenol is one of the crucial products in cinnamon and clove trees [38]. It possesses various antioxidant, anti-inflammatory, anticancer, and antimicrobial properties [39]. Eugenol has numerous uses because of its diverse biological actions [40, 41]. Considering that the cerebellum plays vital roles in motor learning, motor activities and balancing, and SNCA/MAPT/LZTS3 have been implicated in neurodegeneration, this study investigates the neurotoxicity effects of PQ and eugenol on SNCA/MAPT/LZTS3 genes in impaired limb functionality, motor activity and balancing of Wistar rats.

## Methods

### Sources of reagents and chemicals

Paraquat was purchased from Sigma Aldrich with a CAS Registry Number 1910-42-5, melting point 175-180^0^ C, and density/m³ 1.25 g/c with a purity of 99%. Eugenol monomer powder (99.8%) was purchased from Jiangxi Planty Manor Health Industry & Co., Ltd.

### Ethical clearance

The National Institutes of Health’s guidelines for the use and care of animals were adhered to strictly during the experiment. The Alex Ekwueme Federal University Ndufu Alike (AE-FUNAI) Ethics and Animal Handling Committee approved the research, referencing AE-FUNAI-2023/00321.

### Toxicity test

An oral toxicity test to determine the LD_50_ of eugenol was performed according to Lorke’s method with slight modifications. Twenty-five male adult Wistar rats were divided into 5 groups of 5 rats under standard conditions. The rats received 1000 mg/kg, 2000 mg/kg, 3000 mg/kg, 4000 mg/kg, or 5000 mg/kg body weight eugenol orally for seven days. The rats were monitored for signs of toxicity ranging from discomfort to death. At the end of the seven days, none of the rats died, but some discomfort was observed in the 5000 mg/kg group immediately after administration, which soon disappeared.

### Experimental protocol

**Fig. 1:**
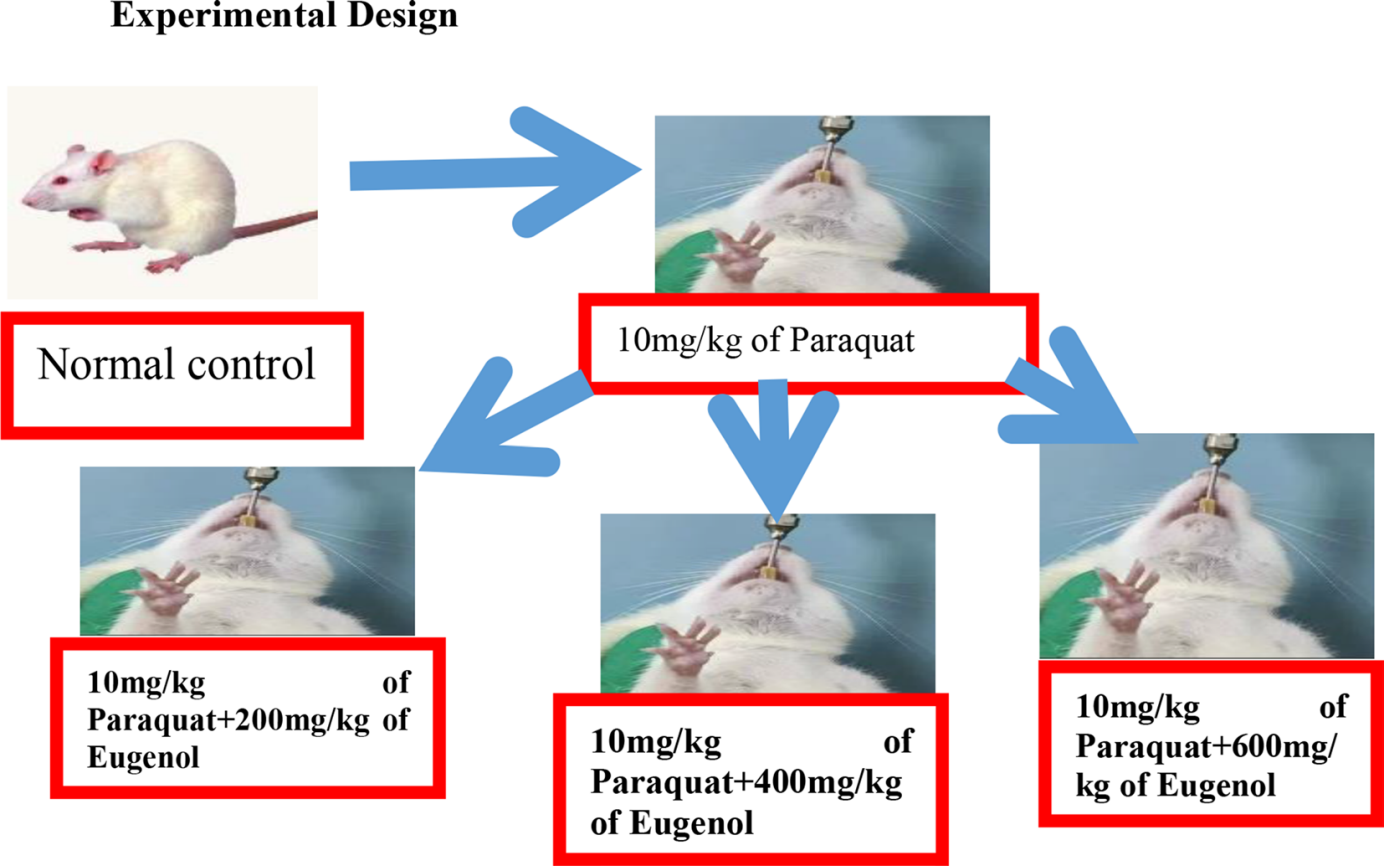
A simple diagram showing the experimental design

The Forty (40) adult male Wistar rats used in the experiment were obtained from the Alex Ekwueme Federal University Ndufu-Alike, Ebonyi State (AE-FUNAI) animal house and acclimatized under standard conditions for fourteen (14) days. After that, the rats were weighed and assigned to five (5) groups of eight (8) rats per group, and weighing was performed once a week. The rats were induced with Paraquat before treating them with eugenol using graded dosages. Group A was the control group and received normal saline using oral gavage in addition to feed and water; group B received 10 mg/kg body weight of Paraquat as the untreated group. Group C received 10 mg/kg of Paraquat and then treated with 200 mg/kg per body weight of eugenol; Group D received 10 mg/kg of Paraquat and, after that, was treated with 400 mg/kg per body weight of eugenol while Group E received 10 mg/kg of Paraquat and after treated with 600 mg/kg per body weight of eugenol. Paraquat was given orally for 14 days at one-day intervals. This procedure allowed the PQ in the rats for 28 days. The eugenol treatment started on the 29th day of the experiment and continuously lasted 14 days. The paraquat induction and eugenol treatment lasted for 42 days before the rats were sacrificed. All administrations were done orally in the morning hours using oral gavage. Figure 1 shows the experimental protocol.

### Assessment of grip strength and limb impairment

The grip strength of the rats was assessed using a steel wire hanger 2 mm in diameter and 60 cm in length, and the rats were allowed to hold on to the wire with their forepaws and were placed 50 cm high over a piece of foam. The time taken and the latency to release the paws and fall were recorded with a cut-off time of 180 s. The grip loss latency was considered a measure of grip strength according to the method of Jasson et al. [42]. Limb impairment was assessed according to the methods of [42] in which rats were scored 3 for gripping the wire with both hind paws, 2 for gripping the wire with one hind paw, and 0 for not gripping the wire with either of the hind paws. The results are expressed as the mean of the total animal scores per group, and the experiment was conducted on the 40th day of the experiment.

### Estimation of motor coordination and balance

The beam work test protocol was used to determine the rats’ fine motor coordination and balance, particularly for detecting subtle deficits in motor skills and balance that other motor tests may not detect [43]. The Wistar rats were trained two days before testing, according to Southwell et al. [44]. The beam work can detect motor impairments due to brain lesions and genetic and pharmacological effects. The balance beam test requires using a narrow beam with varying dimensions elevated from the ground [45]. The start end is made of avoidance stimuli, while the finish end has a goal box serving as an escape from the stimuli. The animals were placed on an elevated wooden beam and allowed to cross into a wooden goal box. The beams were 2.5 cm by 1 m wide and high, respectively. Three trials were conducted for each session in which the latency to traverse the beam and the number of hind slips were observed, and the mean score was recorded using a grading system [46, 47]. Rats typically walk with their feet flat on the surface of the beam, and before the experiment, the apparatus was cleaned at intervals to prevent the influence of lingering stimuli. The experiment was video-tracked, and at the end, the following parameters were collected and analyzed: latency to initiate the task, latency to cross beam, hind leg foot slips, number of falls during the trial, and the total number of steps [48]. The beam walk for studying balance and equilibrium was conducted on day 41 of the experiment.

### Animal sacrifice

The rats were sacrificed by cervical dislocation after the study. The skull was excised, and the cerebellum was harvested. Some cerebellums were fixed in 10% formal saline for histological studies, some were homogenized for biochemical assays, while others were frozen and used to analyze relative gene expression. The homogenate was centrifuged for 10 min at 4,000 rpm at 4 °C, and the supernatants were frozen at approximately − 4 °C.

### Estimation of reactive oxygen species (ROS)

ROS were measured using ROS Nalondi kits (Product Code: NS-15022, Navandsalamat Co.). In brief, kit sinks were covered with an initial antibody against the ROS antigen. Then, the ROS antigen was added, followed by a secondary antibody bound to the antigen. The enzyme in HRP was subsequently added, and the secondary antibody was added. Washing was performed, and chromogenic solutions containing the HRP enzyme substrate were added. Oxygenated water (H_2_O_2_) was exposed to HRP and converted into hydroxide radicals, which were subsequently exposed to a reagent that oxidized and produced colour. The addition of a sulfuric acid solution stopped the production of colour. An ELISA Reader was used to study the colour. The optical density (OD) and the sample concentration were determined based on the colour—the more colour there was, the greater the absorbance.

### Estimation of antioxidant activities

Superoxide Dismutase (SOD) was measured based on its ability to inhibit the reduction of nitro-blue tetrazolium (NBT). The mixture constituted of 2.7 ml of 0.067 M phosphate buffer, pH 7.8, 0.05 ml of 0.12 mM riboflavin, 0.1 ml of 1.5 mM NBT, 0.05 ml of 0.01 M methionine, and 0.1 ml of enzyme. The tubes were uniformly illuminated by placing them in the air with aluminum foil in a box with a 15 W fluorescent lamp for 10 min. A control without the enzyme source was included, and the absorbance was read at 560 nm. One unit of SOD equals the amount of enzyme needed to inhibit the reduction of NBT by 50% under specific conditions. The amount of GPx was determined using a Ransel kit made by Randox Laboratories Ltd., Crumlin, UK, by measuring the oxidation rate of NADPH at 340 nm. A unit of the enzyme was shown as the amount of enzyme required to oxidize 1 nmol of NADPH oxidase/minute. 5,5’-Dithibios (2-nitrobenzoic acid) (0.1 mmol/L) in a 0.1 mol/L disodium phosphate buffer solution was titrated to a pH of 8. The reduced glutathione content was measured at 412 nm, and the reduced product, thionitrobenzene, was quantified spectrophotometrically. The GSH concentration is given as mol/g of tissue.

### Gene expression study

#### Isolation of total RNA

Tissue samples were treated with the Quick-RNA MiniPrep™ Kit from Zymo Research to isolate the total RNA. The DNA contaminants were removed following treatment with DNAse I (NEB, Cat: M0303S). A spectrophotometer from the A&E Lab was used. In the UK, RNA was measured at 260 nm, and the purity was verified at 260 and 280 nm [49].

#### cDNA conversion

One (1 µg) of DNA-free RNA was converted to cDNA by reverse transcriptase reaction with the aid of a cDNA synthesis kit based on ProtoScript II first-strand technology (New England Biolabs) in a 3-step reaction for 5 min at 65 °C, an hour at 42 °C, and 5 min at 80 °C [50].

#### PCR amplification and agarose gel electrophoresis

The amplification of the gene with polymerase chain reaction (PCR) was carried out with OneTaqR2X Master Mix (NEB) using the following primers (Inqaba Biotec, Hatfield, South Africa). PCR amplification was done in a 25 µl volume reaction mixture containing cDNA, primers (forward and reverse), and Ready Mix Taq PCR master mix. Under these conditions, initial denaturation was performed within 5 min at 95 °C, followed by 30 cycles of amplification (30s denaturation at 95 °C, 30s of annealing, and extended at 72 °C for 60s) and a final extension of 10 min at 72 °C [49, 50]. 1.0% agarose gel was used to resolve the amplicon. The GAPDH gene was used to normalize each gene’s relative expression level, and band intensity was quantified using ImageJ software [51].

### Primer sequence

A primer sequence is described as short nucleotide sequence that serves as a starting point for DN synthesis, primarily during polymerase chain reaction, as represented in Table 1 below.

**Table 1:** The table showing the primer sequence of the gene expression

	LZTS3	MAPT	SNCA	GAPDH
Forward	‘5-CGCTGCTCAGCTCTGATTAT-3’	‘5-CAGATGCCTGCCTGAGAAA-3’	‘5-GATGGGCAAGGGTGAAGAA-3’	‘5-CTACCTTCCTTCTGCCCAATAC-3’
Reverse	‘5-CAGGGAAACACAGGAAGACA-3’	‘5-CTACCTTCCTTCTGCCCAATAC-3’	‘5-CAGGTTCATAGTCTTGGTAGCC-3’	‘5-CATCGTACTCCTGCTTGCTG-3’

### Image quantification

ImageJ software version 1.53j was used to quantify both the relative gene expression of SNCA, MAPT, and LZTS3; and the photomicrographic plates of the cerebellum. The data are expressed in figures, analyzed, and presented in charts.

### Data analysis

The data were analyzed using GraphPad Prism version 8.0.1. The means were compared using a Two-way analysis of variance (ANOVA), a significance level of *p* < 0.01 was established, and a standard error of the mean (SEM) was demonstrated in the charts. The means were compared using the Turkey multiple comparison test.

## Results

### Effects of PQ and eugenol exposure on weight

**Fig. 2:**
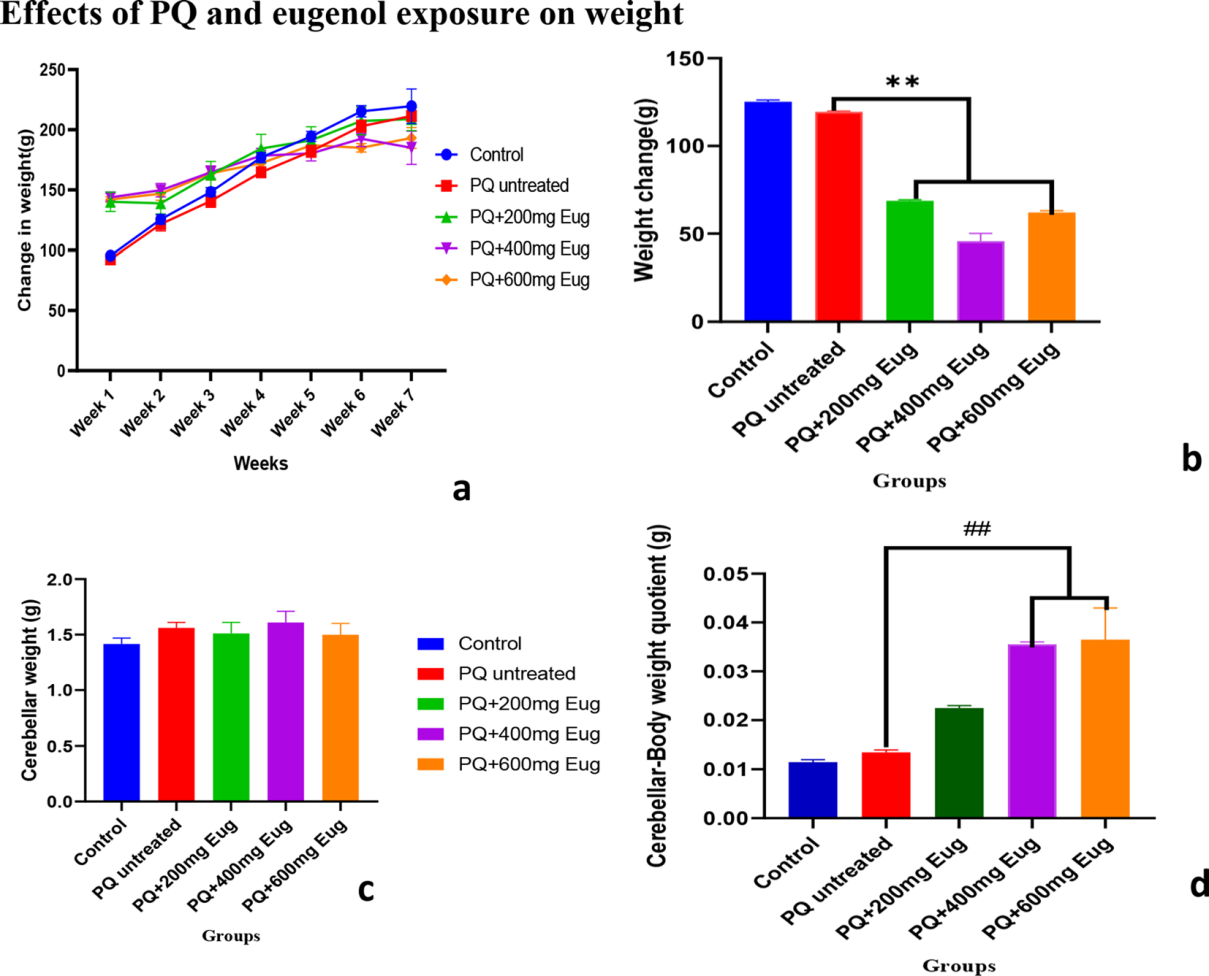
The effects of paraquat administration and eugenol treatment on weight during the experimental period. (**a**) Weight of the animals by week; (**b**) weight change; (**c**) brain weight; (**d**) weight change-brain quotient. **Significant decrease compared to the PQ-untreated group at *p* < 0.05; ##Significant increase compared to the PQ-untreated group (*p* < 0.05)

The weights were calculated and are presented in Fig. 2a - d. The figures show the effects of paraquat intoxication and eugenol treatment on the animal and brain weights. Figure 2a shows body weight from week one to week seven of the experiment. The results indicated that the weights increased from week one to the last week of the experiment. Figure 2b represents the relative weight change from week one to week seven. There was a significant weight reduction in the eugenol-treated groups compared to the PQ-untreated group (*p* < 0.01). Figure 2c shows the brain weights of the animals, and there was no significant difference between the groups. Figure 2d shows the relative brain weight calculated by dividing the brain weight (BW) by the change in weight (CW). The results showed that the body weight-brain quotient of the eugenol-treated group was significantly greater than that of the untreated group (*p* < 0.01).

### Muscle tone and limb functionality

**Fig. 3:**
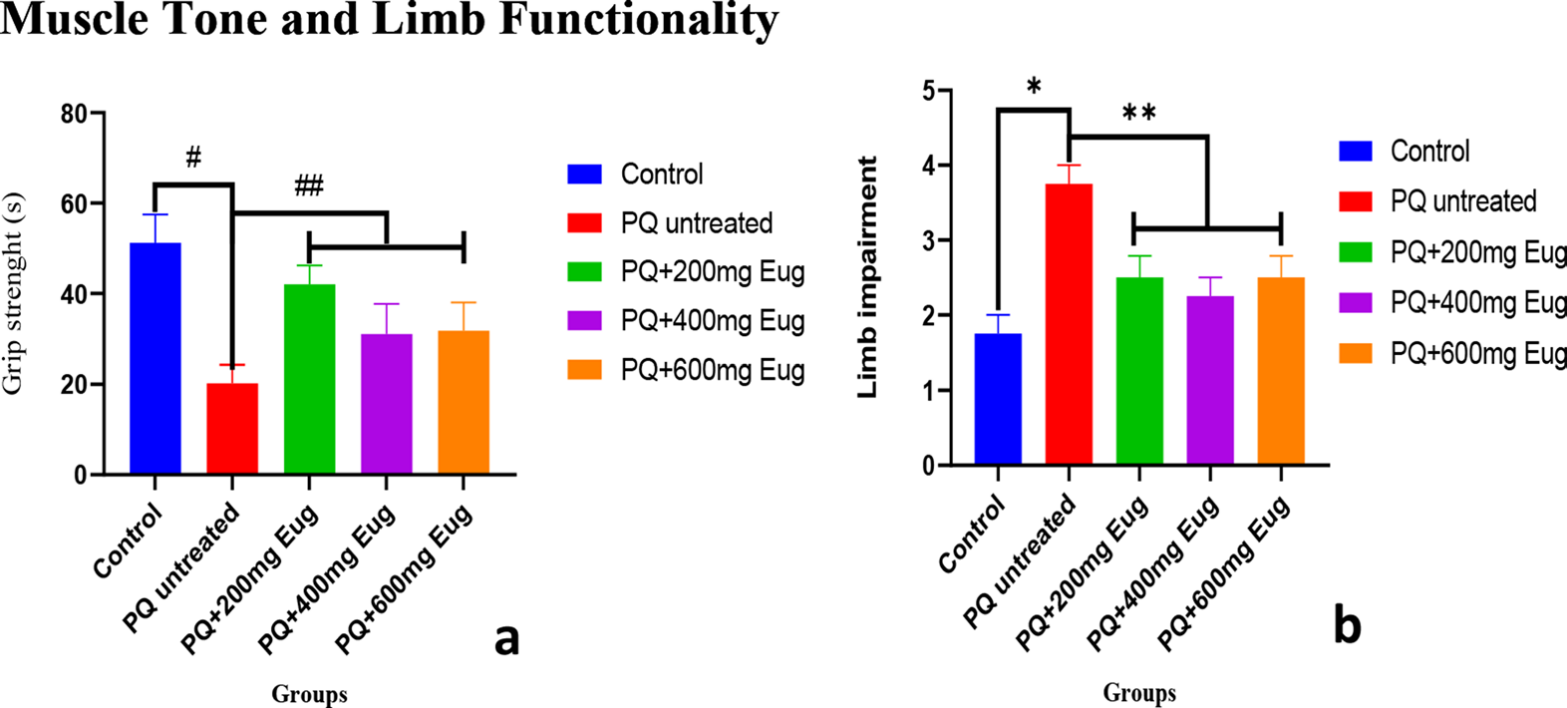
The effects of paraquat administration and eugenol treatment on muscle tone and limb functionality on (**a**) grip strength and (**b**) limb impairment. *Significant increase compared to the control group (*p* < 0.05); **Significant decrease compared to the PQ-untreated group (*p* < 0.05); #Significant decrease compared to the control group (*p* < 0.05) and ##Significant increase compared to the PQ-untreated group (*p* < 0.05)

Muscle tone and limb functionality are used to study rats’ grip strength and limb impairment. Figure 3a and b show the animals’ grip strength and limb impairment. Grip strength was significantly lower in the PQ-untreated group than in the control group (*p* < 0.01). Compared with those in the untreated group, the grip strength in the 200 mg/kg, 400 mg/kg, and 600 mg/kg eugenol treatment groups significantly increased (*p* < 0.01) (Fig. 3a). The results of the limb functionality tests are shown in Fig. 3b, with the untreated group showing significantly more significant limb function impairment than the control group (*p* < 0.01). Compared with those in the untreated group, the limb impairment in the eugenol-treated group significantly decreased (*p* < 0.01).

### Motor activity and balance

**Fig. 4:**
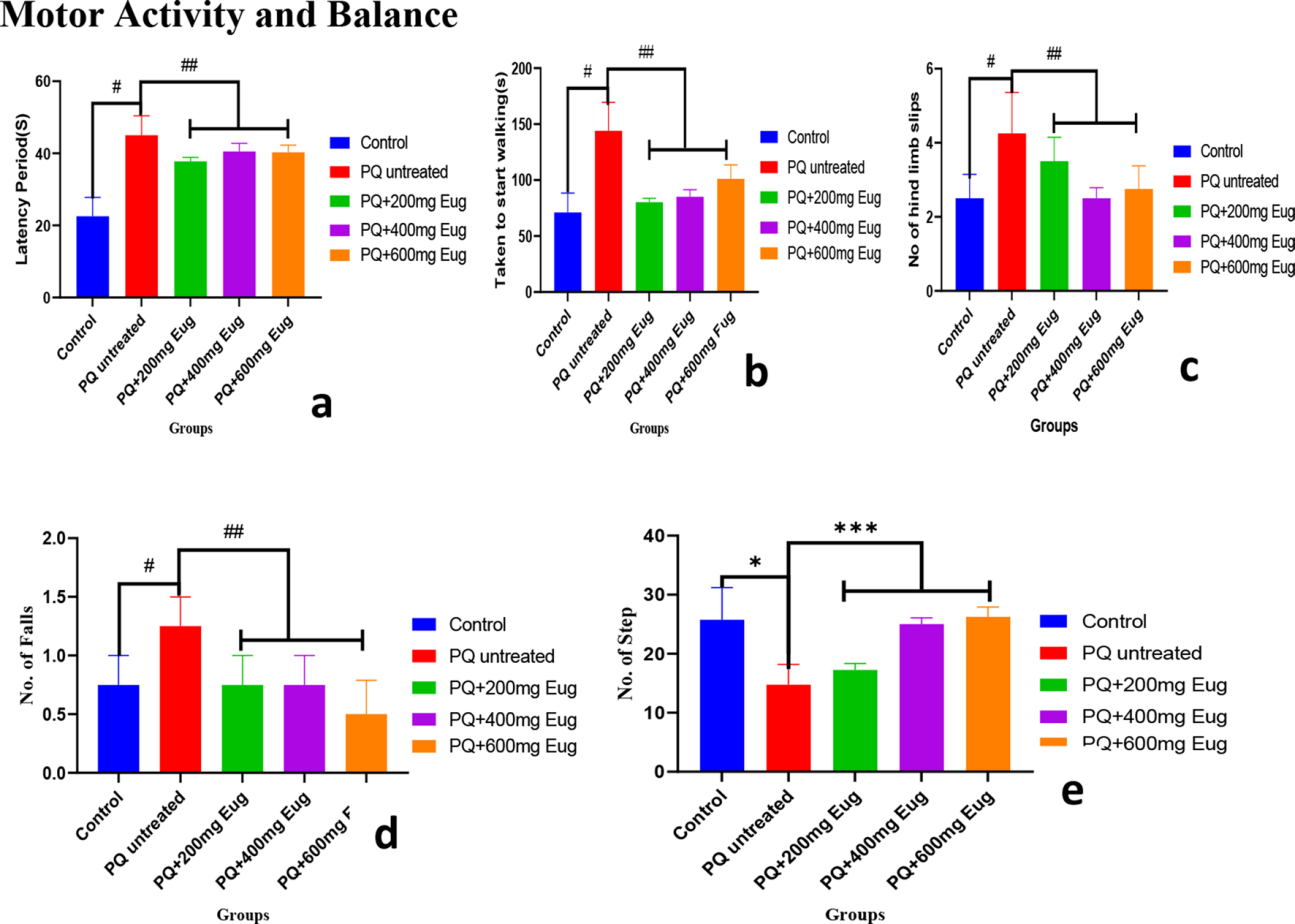
The effects of paraquat administration and eugenol treatment on muscle tone and limb functionality according to the beam walk test: (**a**) latency, (**b**) time to start walking, (**c**) number of hind limb slips, (**d**) number of falls, and (**e**) total number of steps. *Significant increase compared to the control group (*p* < 0.01); **Significant decrease compared to the PQ-untreated group (*p* < 0.01); #Significant decrease compared to the control group (*p* < 0.01) and ##Significant increase compared to the PQ-untreated group (*p* < 0.01)

The results in Fig. 4a-e represent the results of the beam walk used to measure motor activities and limb functionality. The latency, time to start walking, number of hind limb slips, and number of falls were significantly more significant in the untreated PQ group than in the control group (*p* < 0.01) (Fig. 4a-d). The time at which the rats started to move, the latency period, the total number of falls, and the number of hind limb slips were significantly lower in the eugenol-treated groups than in the PQ-untreated group (*p* < 0.01) (Fig. 4a-d). The total number of steps performed by the rats during the experiment was significantly lower in the PQ-untreated group than in the control group (*p* < 0.01). The number of steps increased significantly in a dose-dependent manner in the eugenol-treated groups compared to the PQ-untreated group (*p* < 0.01), as shown in Fig. 4e.

### Antioxidant activities

**Fig. 5:**
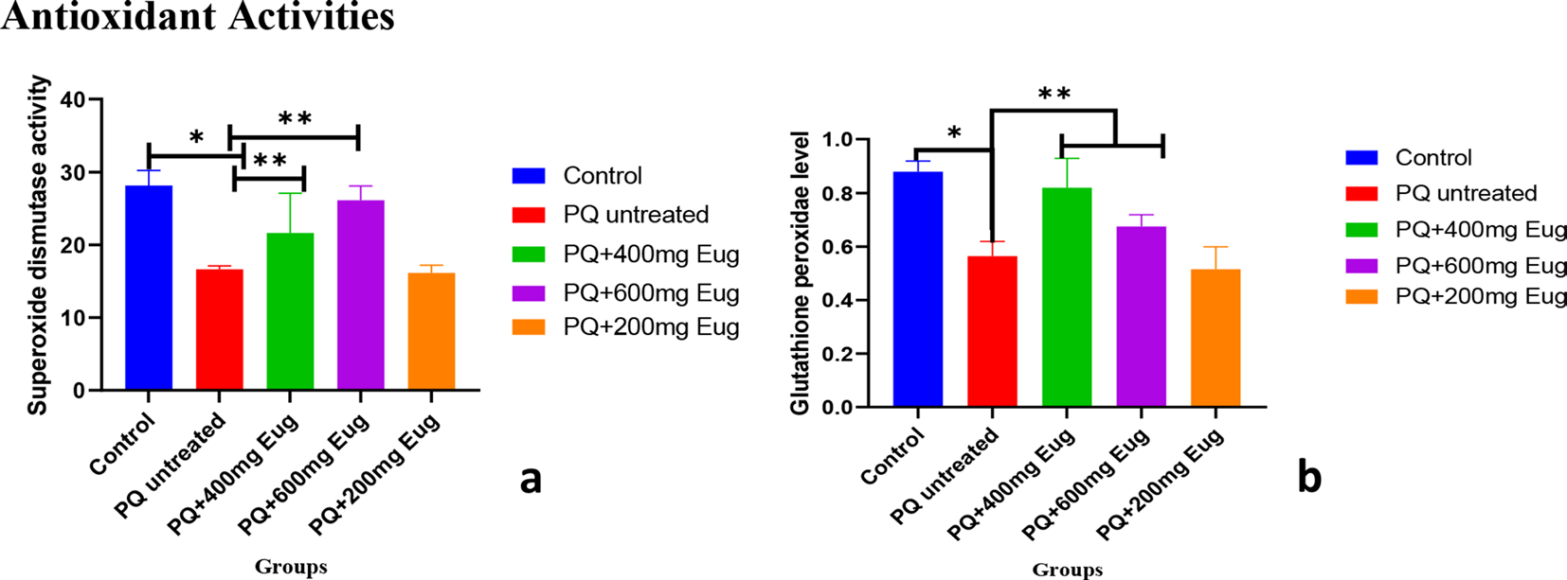
The effects of paraquat administration and eugenol treatment on antioxidant levels. (**a**) Superoxide dismutase (SOD) activity and (**b**) glutathione peroxidase (GPx) activity. *Significant decrease compared to the control group (A) (*p* < 0.01); **Significant increase compared to the untreated group (*p* < 0.01)

Antioxidants have been shown to neutralize free radicals generated within biological tissues, adversely affecting organisms. The antioxidant enzymes measured in the present study are shown in Fig. 5a and b. The results in Fig. 5a show that the activity level of superoxide dismutase (SOD) was significantly lower in the untreated group than in the control group (*p* < 0.01). The results also showed that the SOD activity level was significantly greater in the low- and medium-dose groups than in the untreated PQ group (*p* < 0.01). The level of glutathione peroxidase (GPx) shown in Fig. 5b was significantly lower in the paraquat-treated group than in the control group (*p* < 0.01). GPx was significantly greater in the low and medium-dose groups than in the PQ-untreated group (*p* < 0.01).

### Oxidative stress markers

**Fig. 6:**
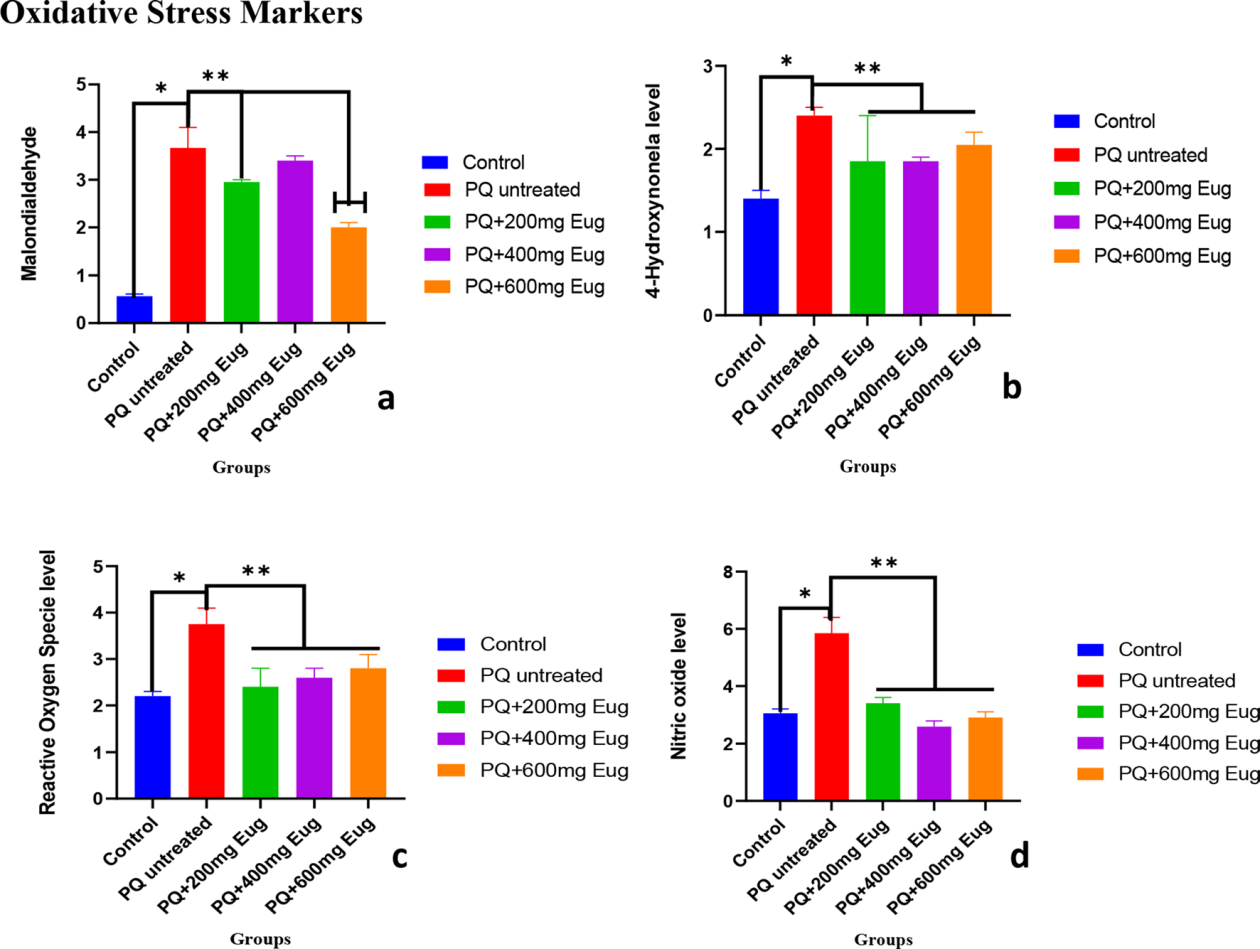
The effects of paraquat administration and eugenol treatment on oxidative stress markers. (**a**) Malondialdehyde (MDA), (**b**) 4-hydroxynonenal (4HNE), (**c**) reactive oxygen species (ROS) and (**d**) nitric oxide (NO). *Significant increase compared to the control group (A) (*p* < 0.01) **Significant decrease compared to the untreated group (*p* < 0.01)

Increased oxidative stress leads to increased protein degradation due to elevated reactive oxygen species (ROS) in the system. The level of oxidative stress measured in the present study is shown in Fig. 6a–d. The levels of malondialdehyde (MDA), reactive oxygen species (ROS), 4-hydroxynonenal (4HNL), and nitric oxide (NO) increased significantly in the PQ-untreated group compared to those in the control group (Fig. 6a, b, c and d) (*p* < 0.01). Figure 6a shows that the level of MDA in the low- and medium-dose groups decreased significantly compared to that in the PQ-untreated group (*p* < 0.01). Compared with those in the untreated group, the levels of 4HNL, ROS, and NO in Fig. 6b, c, and d were significantly lower following treatment with various doses of eugenol (*p* < 0.01).

### Relative gene expression

**Fig. 7:**
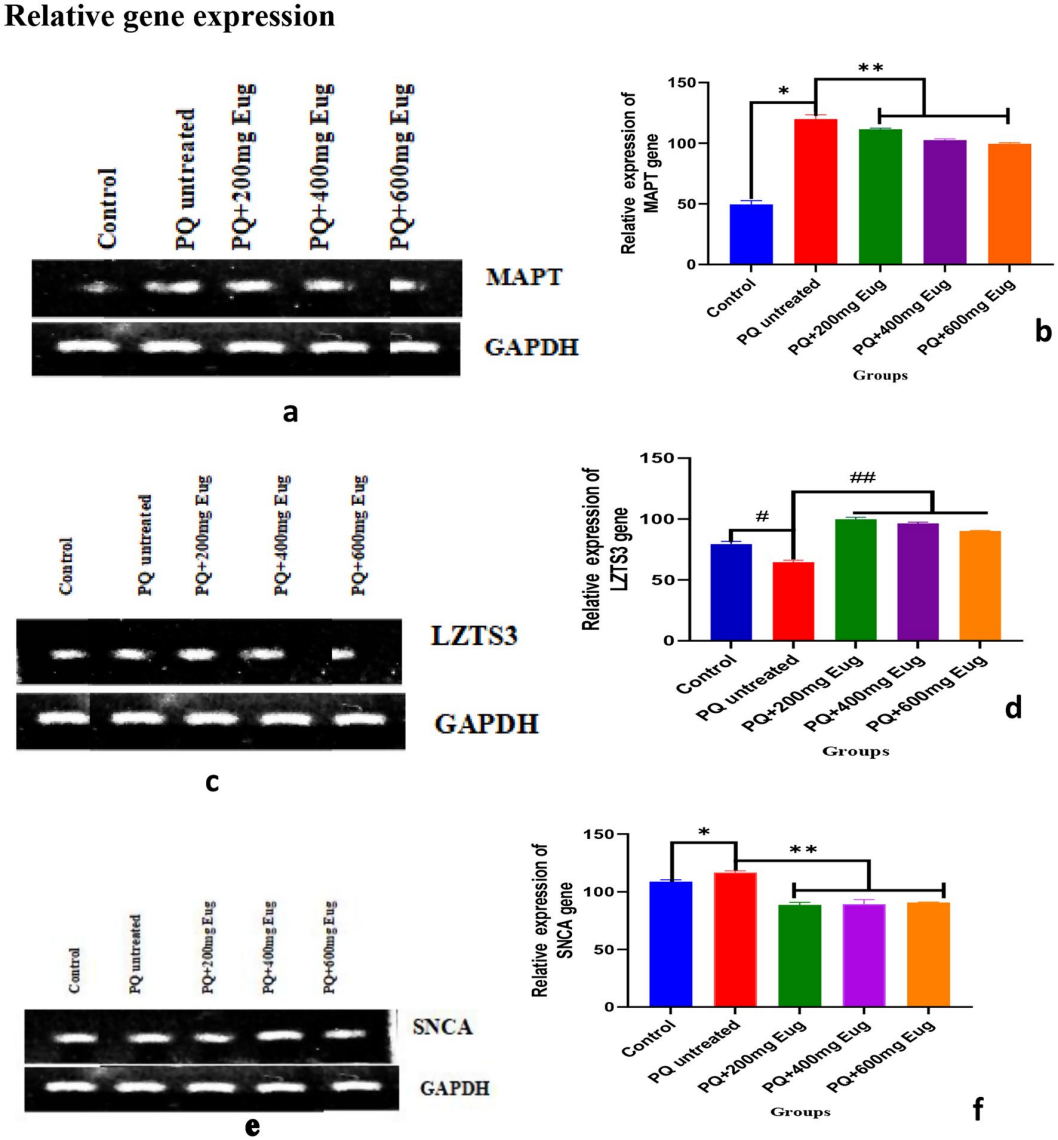
Effect of paraquat administration and eugenol treatment on gene expression in the cerebellum of Wistar rats. (**a**) MAPT gene expression bands, (**b**) relative MAPT gene expression, (**c**) LZTS3 gene expression bands, (**d**) relative LZTS3 gene expression, (**e**) SNCA gene expression bands, and (**f**) relative SNCA gene expression. *Significant increase compared to the control group (A) (*p* < 0.01) and **Significant decrease compared to the PQ-untreated group (*p* < 0.01); #Significant increase compared to the control group (A) (*p* < 0.01) and ##Significant increase compared to the PQ-untreated group (*p* < 0.01)

The relative gene expression results are shown in Fig. 7a-f, and the results in Fig. 7a and c, and 6e represent the MAPT, LZTS3, and SNCA genes, respectively. Gene expression was quantified using ImageJ software, and the data were analyzed and are shown in Fig. 7b and d, and 7f. The results in Fig. 7a, b, e, and f show that the MAPT and SNCA genes are significantly overexpressed compared to those in the control group (*p* < 0.01). The expression of these genes decreased when eugenol was administered to the animals. LZTS3 expression was significantly lower in the PQ-untreated group than in the control group (*p* < 0.01) (Fig. 7c and d). Compared with that in the PQ-untreated group, LZTS3 gene expression in the eugenol-treated group was significantly greater (*p* < 0.01) (Fig. 7c and d).

### Cerebellum histology

**Plate 1:**
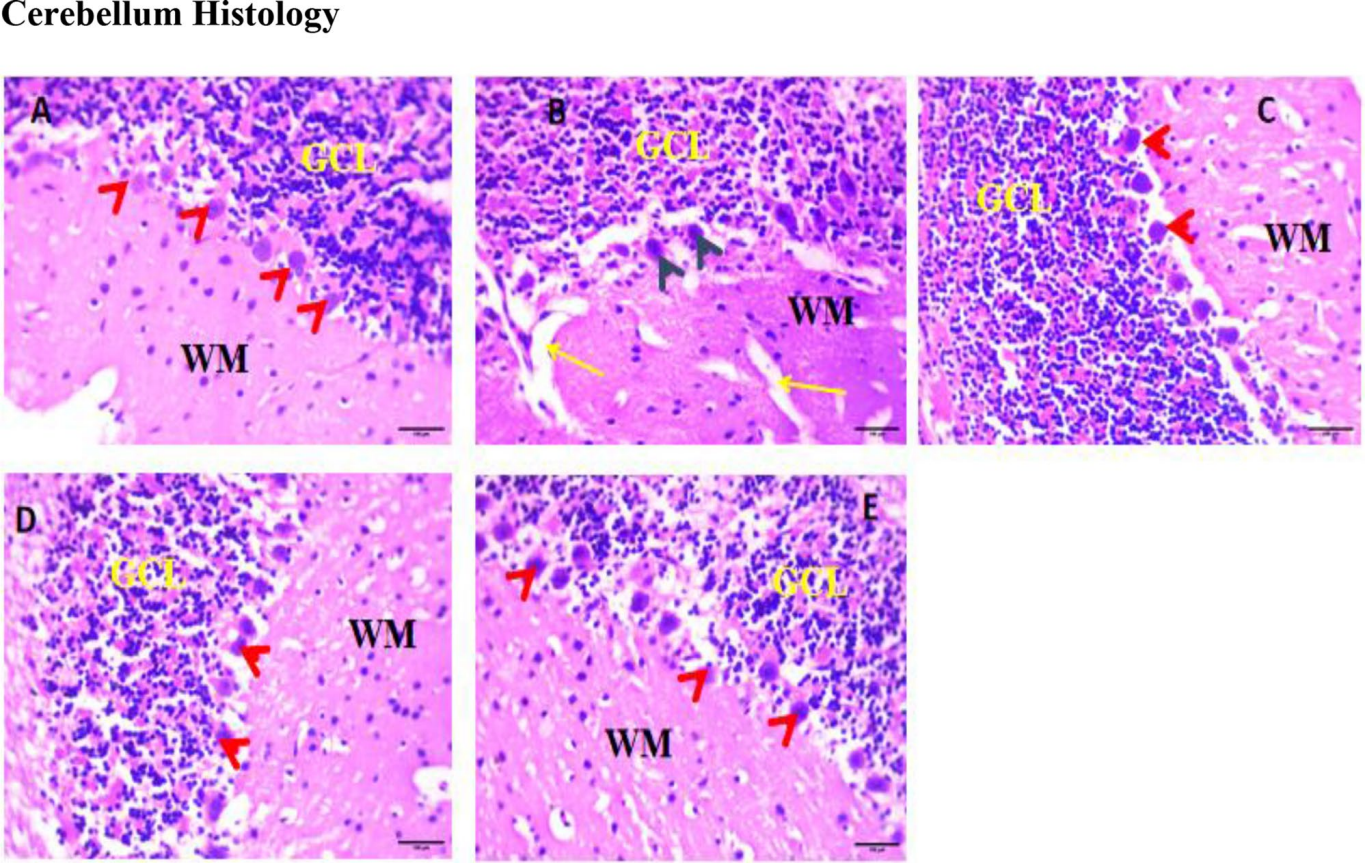
A section of the cerebellar cortex showing granular cell layers (GCLs) and white matter (WM). **A** shows normal Purkinje cells (red arrowheads), **B** shows necrotic nuclei, reduced Purkinje cells (black arrowheads), and vacuolations (yellow arrows), and **C**, **D**, and **E** show normal Purkinje cells (H & E; X400, 100 μm)

**Fig. 8:**
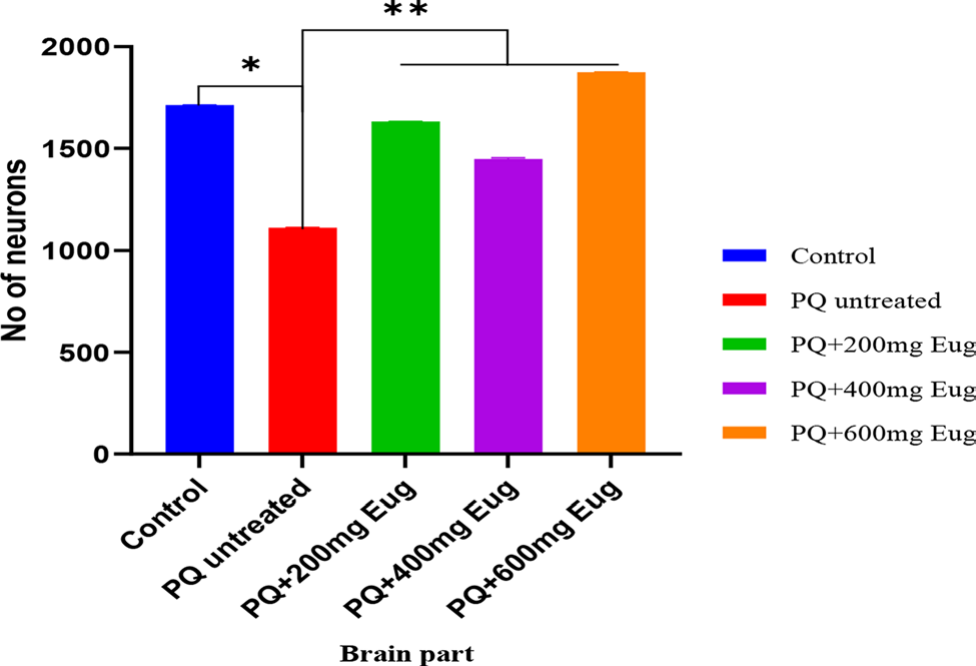
Number of neurons present in sections of the H & E of the cerebellum. *Significant decrease compared to the control group (*p* < 0.01); **Significant increase compared to the PQ-untreated group (*p* < 0.01)

Plate 1 shows a cerebellum section stained with hematoxylin and eosin (H&E). The results in the control group (A) show normal histoarchitecture of the cerebellum with well-arranged Purkinje cells at the border of the granular layer and the white matter. The results showed healthy and well-arranged granular cells on Plate 1 A. The untreated group (B) showed several vacuolations in the granular layer and white matter with reduced Purkinje cells at the border, and few granular cells were observed (Plate 1B). Group C showed improvement compared to the PQ-untreated group, with increased Purkinje and more granular cells (Plate 1 C). The medium- and high-dose groups (D and E) showed healthier cells than the PQ-untreated group (B). Compared with Group B, the Purkinje cells in Plates 1D and 1E were more significant. Compared with those in the control group, the number of neurons in the PQ-untreated group was significantly lower (*p* < 0.01). Compared with those in the PQ-untreated group, the number of neurons in the cerebellum in the eugenol-treated group was significantly greater (*p* < 0.01) (Fig. 8).

### Alpha-synuclein staining

**Plate 2:**
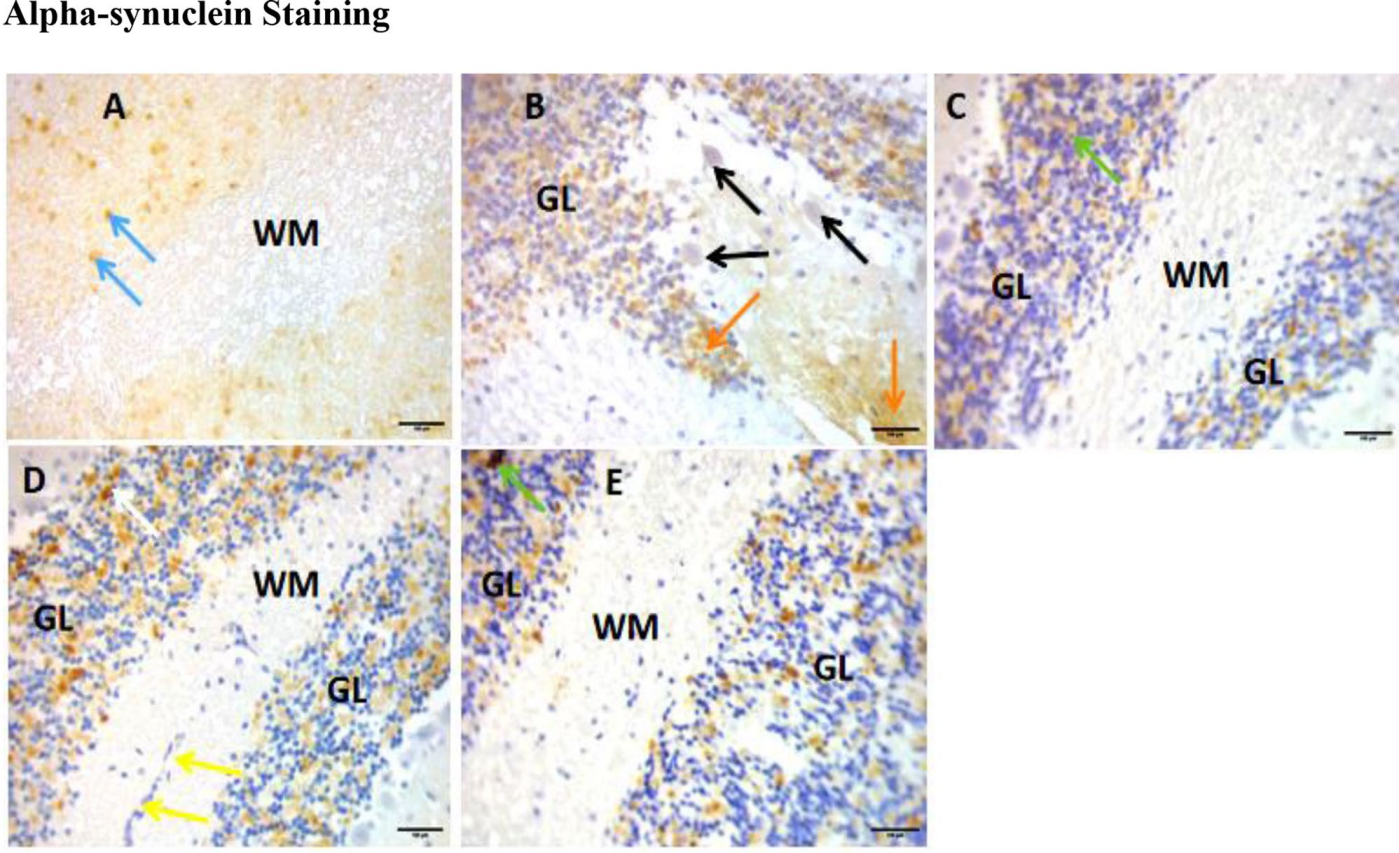
Sections of the cerebellum stained with α-Synuclein showing Lewy bodies scattered throughout the tissue (blue arrows, **A**; red arrows, **B**; green arrows, **C** and **E**; and white arrows, **D**); white matter and granular layer. X400 and 100 μm

**Fig. 9:**
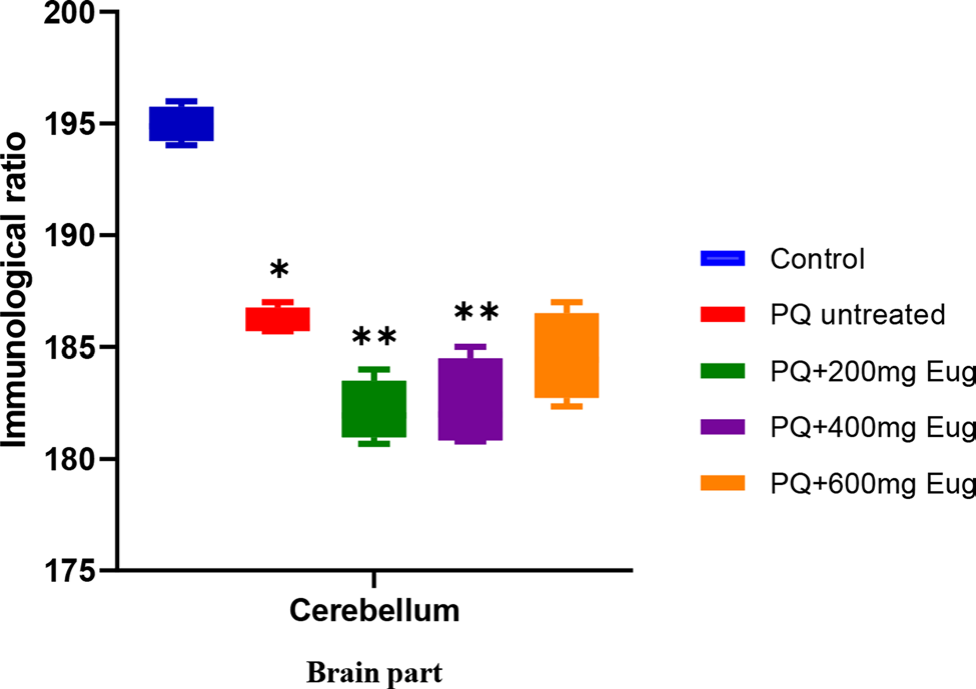
Immunological ratio of cerebellar sections stained with α-Synuclein. *Significant decrease compared to the control group (*p* < 0.01); **Significant decrease compared to the PQ-untreated group (*p* < 0.01)

In Plate 2, α-Synuclein was highly expressed in the control group (A). The results showed the presence of alpha-synuclein in the cerebellum with Lewy bodies. The paraquat-untreated group (B) exhibited different staining than the control group (A), with many more Lewy bodies. The paraquat-untreated group (B) and the eugenol-treated groups (C, D, and E) expressed more alpha-synuclein than the control group. The alpha-synuclein did not stain the Purkinje cells, as shown in the micrographs. Figure 9 shows the immunological ratio of α-Synuclein expression in the cerebellum. The expression was significantly lower in the PQ-untreated group than in the control group (*p* < 0.01). There was a significant decrease in α-Synoclein expression in the treated groups compared to that in the PQ-untreated group (*p* < 0.01) (Fig. 9).

### NeuN staining

**Plate 3:**
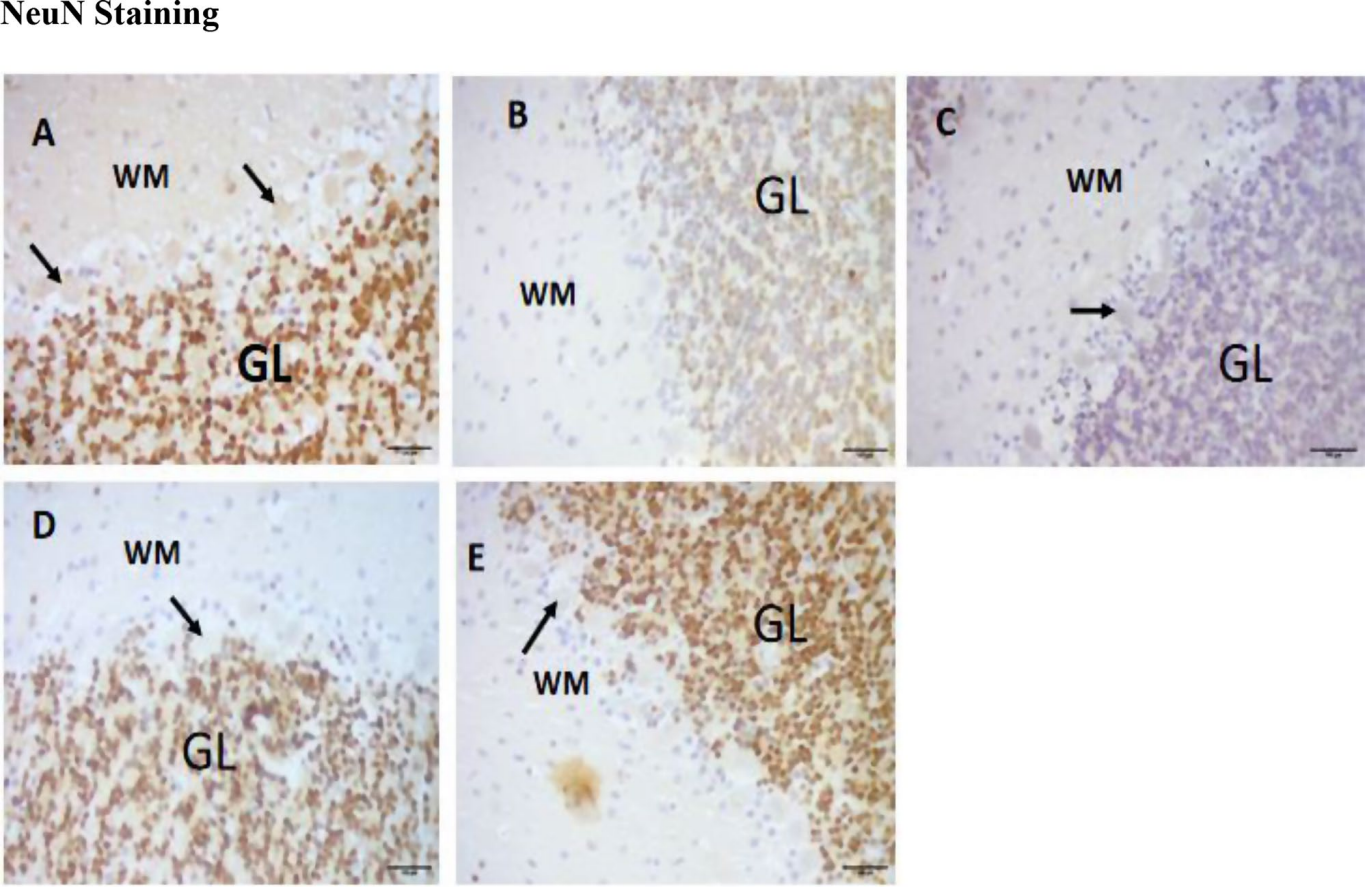
Sections of the dorsal part of the cerebellum showing normal histology and healthy cells in A. B shows no NeuN staining or necrosis. C shows many mature and normal neuronal cells (blue arrow). D and E appeared normal, with several mature neurons. Other groups show several Purkinje cells (black arrows). X400 and 100 μm

**Fig. 10:**
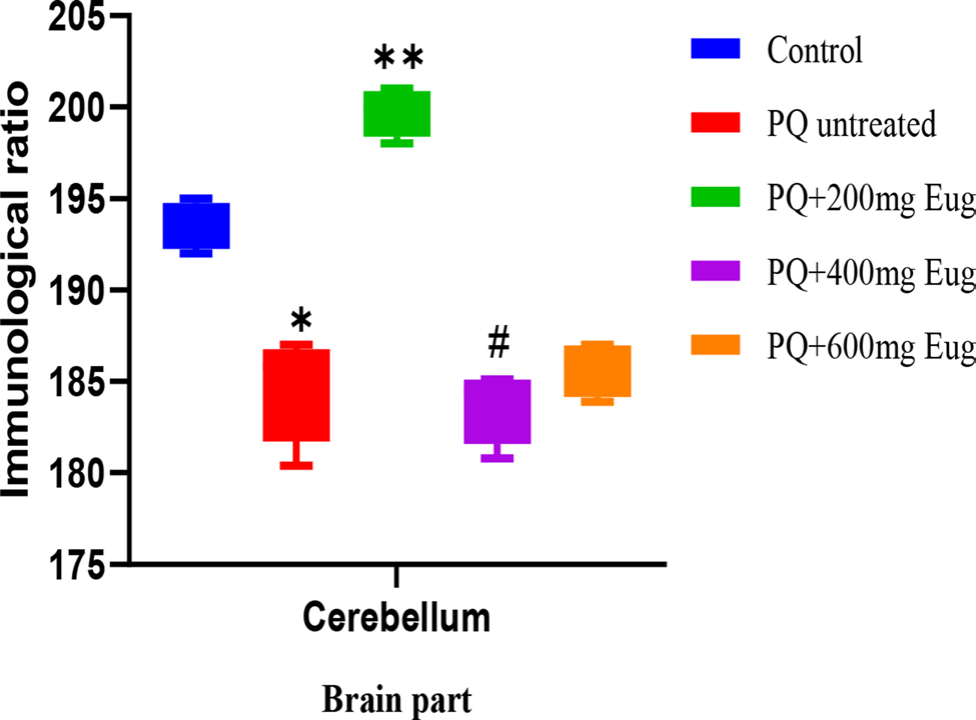
Immunological ratio of NeuN-stained cerebellar sections. *Significant decrease compared to the control group (*p* < 0.01); **Significant increase compared to the PQ-untreated group (*p* < 0.01); #Significant decrease compared to the PQ-untreated group (*p* < 0.01)

Sections of the cerebellum stained with NeuN are shown on Plate 3. The results show that the control (A) group exhibited high expression of NeuN, with several Purkinje cells lining the borders. The results of the paraquat-untreated (B) group showed little evidence of NeuN expression, with fewer Purkinje cells at the borderline. The eugenol-treated groups (C, D, E) exhibited more significant NeuN staining, indicating more mature neurons. The low-dose (C) group expressed more NeuN than any other group. Figure 10 shows that NeuN expression was significantly lower in the PQ-untreated group than in the PQ-untreated group. NeuN expression was more significant in the low-dose group than in the PQ-untreated (B) group.

## Discussion

Due to its neurotoxicity, which modifies the histoarchitecture of multiple brain regions, including the cerebellum, and consequent behavioural changes, paraquat exposure is one of the most often used models to investigate the mechanism of Parkinson’s disease-related cell death [30]. The findings of this study point to a number of changes in the cerebellar neurons. The decrease in percentage change in animal weight is implied to be due to the paraquat neurotoxicity, which was improved significantly by the eugenol treatment as a mark of its therapeutic property. The current findings run counter to those of Nathalia et al. [52] and Li et al. [53], who claimed that rats exposed to 10 mg/kg of PQ for five days did not experience any changes in body weight but instead displayed cognitive, learning, and memory impairments. The disparity recorded in weight loss could be due to the length of the study, as our study is a sub-chronic study, while the studies cited above-concerning weight are acute studies. Sub-chronic studies of neurotoxicity caused about 10–15% brain weight loss and between 20 and 30% body weight loss [54]. Weight loss does not just happen; it is an accumulation of several factors, such as elevated ROS, oxidative stress, free radicals, inflammatory cytokines, and reduced antioxidant levels, which would have affected the genetic makeup of such an organism.

Accurate molecular diagnosis and understanding of genetic pathways underlying various neurological and mental illnesses have advanced significantly within the past 20 years. One of the most common comorbidities of neurotoxicity is motor function impairment [47, 55]. Animals were tested using different methodologies to identify the motor deficits caused by neurodegeneration or traumatic brain injury (TBI), according to Sweis et al. [47]. The current research assessed the motor functions of rats using the wire string method and beam walk test. The significant decrease in grip strength in the PQ-untreated group confirmed a loss of neurons in the brain’s motor areas caused by PQ-induced neurotoxicity [56]. The neurological disorder must have led to muscle stiffness, one of the manifestations of limb impairments [57]. In the treated groups, grip strength increased significantly, indicating that eugenol enhances motor activity.

Furthermore, limb impairment is another parameter that can be measured by the wire hang method to measure motor deficits [58]. There was a significant increase in limb impairment in the rats in the PQ-untreated group, which may have been due to neuronal loss in the brain’s motor centres [59]. The increase in limb impairment corroborates the reduction in the grip strength of the rats [60]. The two-limb functionality works hand in hand, as a reduction in one leads to an increase in the other. Limb impairment may be due to neuronal loss in the cerebellum of the rats. The cerebellum is one of the motor centres of the brain whose neuronal degeneration is capable of causing motor deficits [61]. The eugenol-treated groups showed significantly reduced limb impairment. The improvement recorded in the experiment might be due to eugenol’s antioxidant and anti-inflammatory properties [37].

In the beam test, unsteady walking, slow movement, imbalance, and loss of coordination indicate rodent motor deficits. The parameters measured during the beam walk test were used to assess the motor functionality of the rats. There was a significant increase in the number of falls, time taken to start a movement, number of hind limb slips, and latency period in the paraquat-untreated group. This result may have been caused by the neurodegenerative effects of Paraquat, a substance that has been implicated in impaired motor function [7]. There was also a significant reduction in the total number of steps the rats took in the PQ-untreated group [48]. The eugenol-treated group exhibited significantly decreased time spent traversing the beam, latency period, hind limb slip, and number of falls. The total number of steps the rats took in the treated group was significantly greater than in the PQ-untreated group. These results suggested the therapeutic benefits of eugenol against PQ toxicity.

Gene mutations, specific treatments, and neurodegeneration can alter motor abilities in rats, and the beam walk test assesses motor coordination and balance. The high expression of the SNCA and MAPT genes and reduced expression of LZTS3 in the PQ-untreated group may be attributed to the increase in reactive oxygen species (ROS), malondialdehyde (MDA), 4-hydroxynonela (4HNL) and free radical (NO) in the PQ-untreated group. The increase in the parameters led to a gene mutation that caused gene missense mutations, which, in other words, caused neuronal death. An increase in oxidative stress has been implicated in reduced motor activity, according to Ahn et al. [62]. This finding agrees with the results of the present research. There was a reduction in the antioxidant levels in the PQ-untreated group. This factor may have contributed to the high expression of the SNCA and MAPT genes and led to motor impairment due to neurodegeneration. SNCA, MAPT, and LZTS3 expression increased in response to eugenol treatment, which may be attributed to the significant reductions in ROS, MDA, 4HNL, and NO levels. The reduction in oxidative stress significantly increased the levels of antioxidants, which helped to scavenge the ROS in the system.

## Conclusions

Paraquat is an environmental neurotoxicant that can alter the SNCA, MAPT, and LZTS3 genes by increasing rats’ ROS, free radicals, and oxidative stress levels. Increased oxidative stress causes neurodegeneration in the cerebellum, leading to altered motor activities. Eugenol has been shown to mitigate the effects of Paraquat in rats by reducing ROS, free radicals, and oxidative stress, as well as increasing the level of antioxidants.

## Data Availability

The datasets used and analysed during the current study are available from the corresponding author on reasonable request.

## Abbreviations

4HNL: 4-hydroxynonenal
AE-FUNAI: Alex Ekwueme Federal University Ndufu Alike
ANOVA: Analysis of variance
BW: Brain weight
cDNA: Converted Deoxyribonucleic Acid
CW: Change in weight
DMD: Duchenne muscular dystrophy
DNA: Deoxyribonucleic Acid
GCL: Granular cell layers
GPx: Glutathione Peroxidase
GSH: Glutathione S-Transferase
H & E: Hematoxylin and Eosin
H_2_ O_2_: Oxygenated water
HRP: Horseradish peroxidase
LD50: Lethal Dose
LZTS3: Leucine Zipper Tumour Suppressor 3
MAPT: Microtubule Associated Protein Tau
MDA: Malondialdehyde
NADPH: Phosphorylated Nicotinamide adenine dinucleotide
NBT: Nitro-blue tetrazolium
NO: Nitric oxide
OD: Optical Density
PCR: Polymerase chain reaction
PQ: Paraquat
RNA: Ribonucleic Acid
ROS: Reactive Oxygen Species
SNCA: Alpha-Synuclein
SOD: Superoxide Dismutase
TBI: Traumatic brain injury
WM: White matter
